# Fetal and neonatal alloimmune thrombocytopenia (FNAIT): Survey of UK fetal medicine centres on antenatal management of subsequent affected pregnancies

**DOI:** 10.1111/bjh.70564

**Published:** 2026-05-19

**Authors:** Asha Aggarwal, Catherine Prodger, Brian Hockley, Will Thomas, Mike F. Murphy, Lani Lieberman, James B. Bussel, Evangelia Vlachodimitropoulou, Michael J. R. Desborough

**Affiliations:** ^1^ Department of Clinical Haematology Churchill Hospital Oxford UK; ^2^ NHS Blood and Transplant John Radcliffe Hospital UK; ^3^ Radcliffe Department of Medicine University of Oxford Oxford UK; ^4^ NHS Blood and Transplant Sheffield Blood Centre Sheffield UK; ^5^ Department of Haematology Addenbrooke's Hospital Cambridge UK; ^6^ Department of Laboratory Medicine and Pathobiology University of Toronto Toronto Ontario Canada; ^7^ Weill Cornell Medicine New York USA; ^8^ Maternal Fetal Medicine King's College Hospital London UK; ^9^ Ontario Fetal Center Mount Sinai Hospital, University of Toronto Toronto Ontario Canada

**Keywords:** FcRn, fetal haematology, HPA1a, intravenous immunoglobulin, platelets, pregnancy

## Abstract

Fetal and neonatal alloimmune thrombocytopenia (FNAIT) is a rare condition associated with severe fetal thrombocytopenia and intracerebral haemorrhage. Antenatal management of subsequent pregnancies relies on intravenous immunoglobulin (IVIg); yet, practice varies internationally and UK data are limited. We conducted a national survey to characterise the current antenatal management of FNAIT across UK regional fetal medicine centres using an electronic, scenario‐based design. Responses were analysed descriptively, with ≥75% concordance considered strong agreement. Sixteen of 22 regional fetal medicine centres (73%) responded; all respondents were fetal medicine specialists or haematologists. There was strong consensus to treat standard‐ and high‐risk pregnancies associated with anti‐human platelet antigen (HPA)1a or anti‐HPA5b antibodies using weekly IVIg, most commonly at a dose of 1 g/kg/week. Earlier initiation of IVIg was favoured for high‐risk pregnancies, while timing was more variable for standard‐risk cases. Corticosteroids were not routinely used in standard‐risk pregnancies but were considered in high‐risk scenarios. Fetal blood sampling and intrauterine platelet transfusion were generally avoided. Considerable variation was observed in delivery planning and in the management of pregnancies at risk without a prior confirmed diagnosis. This survey demonstrates the areas of both consistency and variation in UK practice, highlighting gaps in the evidence base and priorities for future research and guidelines development.

**Abbreviations**
FNAIT: fetal and neonatal alloimmune thrombocytopenia
ICH: intracerebral haemorrhage

## INTRODUCTION

Fetal and neonatal alloimmune thrombocytopenia (FNAIT) occurs when maternal antibodies against paternally inherited fetal platelet antigens cross the placenta, leading to fetal thrombocytopenia and associated haemorrhagic complications. These range from mild mucocutaneous bleeding to major bleeding, including intracerebral haemorrhage, which can cause death or long‐term disability.^1^ Approximately 80% of FNAIT cases are due to anti‐human platelet antigen (HPA)1a antibodies.^2^ Anti‐HPA5b antibodies are the second most commonly detected anti‐HPA antibody; however, as they are present in approximately 2% of healthy pregnant women and occur at a similar frequency in pregnancies investigated for suspected FNAIT, their clinical significance is uncertain.^3^

The incidence of FNAIT is approximately 1 in 1200 live births,^4^ with intracerebral haemorrhage (ICH) occurring in up to 1 in 10 of these cases.^5^ There is currently no antenatal screening programme in the United Kingdom or elsewhere, so neonates are typically diagnosed when symptomatic, presenting with bruising or bleeding, sometimes after a first pregnancy.^6^ Following a case of FNAIT, recurrence in future pregnancies is frequent, with the presence or absence of intracerebral bleeding in previous pregnancies the major determinant of risk. The risk of ICH in a subsequent pregnancy is high if a previous fetus or neonate was affected by ICH. In contrast, where a previous pregnancy was affected by thrombocytopenia alone, the risk of ICH in subsequent pregnancies appears to be lower than earlier estimates of up to 7%.^7^, ^8^, ^9^, ^10^

More recent data support a lower absolute risk in this group. Knightly et al.'s retrospective survey of the patient organisation NAITbabies estimated the risk of ICH in untreated subsequent pregnancies without a history of ICH to be 3.2%,^10^ although this may be influenced by selection bias as more severe cases are likely to be represented in patient support organisations. A Norwegian population‐based study by Ernstsen et al. reported no cases of ICH among 64 untreated subsequent pregnancies without a history of ICH (0%, 95% confidence interval [CI] 0.0%–5.7%).^8^ While this study is limited by sample size, taken together these findings suggest that the risk of ICH in subsequent pregnancies without prior ICH is likely lower than previously thought.

Despite this, in most countries, preventative treatment is administered to the mother during future pregnancies to reduce the risk of ICH further. Treatment is started earlier in pregnancy and/or at higher doses for those with a previous fetus or neonate with ICH.

Antenatal management of women who have had a previous pregnancy affected by FNAIT involves treatment with intravenous immunoglobulin (IVIg) at doses of 0.5–2 g/kg/week, starting at 12–20 weeks of gestation and continuing until delivery, sometimes in combination with steroids.^11^ This practice is supported largely by case series and observational cohorts, along with four randomised controlled trials comparing IVIg‐based regimens (with or without corticosteroids), rather than placebo‐controlled studies.^12^, ^13^, ^14^, ^15^, ^16^

An international consensus guideline including antenatal management of FNAIT was published in 2019,^11^ alongside UK‐relevant guidance in a British Journal of Obstetrics and Gynaecology Scientific Impact Paper,^17^ which may help contextualise the current UK practice. Updated recommendations were subsequently issued via an international Delphi consensus statement in 2025.^18^ Collectively, these publications highlight substantial international variation in antenatal management of FNAIT, with limited data describing the current practice in the United Kingdom. The aim of this survey was to characterise the current management of subsequent FNAIT‐affected pregnancies across regional fetal medicine centres in England.

## METHODS

The survey was devised by the primary authors. It was reviewed by members of the UK FNAIT specialist group with input from specialists in fetal medicine, haematology, paediatrics, neonatology, obstetric medicine and laboratory platelet immunology. The survey was created using SNAP Surveys Version 12 incorporating Snap Desktop and Web Hosting (www.snapsurveys.com).

The survey consisted of organisational queries, followed by seven scenarios (Appendix 1). Each scenario had multiple‐choice questions. Skip logic was utilised to maximise survey focus. Overall confidence in managing pregnant patients with a history of FNAIT was assessed using a 5‐point Likert scale ranging from 1 (low confidence) to 5 (high confidence).

The seven scenarios were:

1. A pregnant patient with anti‐HPA1a antibodies and a previous neonate with FNAIT leading to thrombocytopenia without major bleeding;
2. A pregnant patient with anti‐HPA1a antibodies and a previous neonate with FNAIT leading to thrombocytopenia and ICH;
3. A pregnant patient with anti‐HPA5b antibodies and a previous neonate with FNAIT leading to thrombocytopenia without major bleeding;
4. A pregnant patient with anti‐HPA5b antibodies and a previous neonate with FNAIT leading to thrombocytopenia and ICH;
5. A pregnant patient whose sister had experienced a pregnancy affected by FNAIT;
6. A pregnant patient who had an incidental finding of an anti‐HPA1a antibody when she was a platelet donor;
7. A pregnant patient with a previous pregnancy that had a phenotype consistent with FNAIT but tests for antibodies and maternal–paternal crossmatch showed no evidence of FNAIT.

Multiple‐choice questions asked which tests and what type of management respondents would recommend. Additional questions were posed regarding use of IVIg or prednisolone, including the gestational age when this would start, the doses used and escalation strategies.

In the United Kingdom, regional fetal medicine centres are specialist centres for complex obstetric cases. Pregnant patients with a history of FNAIT from local hospitals should be referred to a regional fetal medicine centre for specialist management.^10^

The survey was circulated electronically to consultants specialising in FNAIT at regional fetal medicine centres across England and Scotland. Distribution was undertaken via the NHS Blood and Transplant consultant network and the specialist FNAIT consultant group, with the aim of identifying the clinicians responsible for managing FNAIT at each centre and ensuring comprehensive geographical representation. The survey was launched in June 2024, and two reminder emails were issued to potential respondents prior to its closure in September 2024.

Where more than one consultant from the same regional fetal medicine centre had completed the survey, respondents from the same centre were asked to reach a consensus on any questions where their answers differed. Responses relating to job role and self‐reported confidence were retained on an individual basis, whereas consensus responses were used for all other organisational and clinical items.

If free‐text responses matched an option from the multiple‐choice list, these were reclassified accordingly by the researchers during data cleaning.

Cases were defined as standard risk if the previous pregnancy affected by FNAIT had no intracerebral bleeding and high risk if intracerebral bleeding had occurred.

Descriptive statistics were reported as proportions, means or medians. Management decisions with ≥75% concordance were considered to have strong agreement. Statements with 50%–74.9% concordance were considered to have moderate agreement. Statements with less than 50% were considered to have low concordance.

## RESULTS

Responses were received from 16 of the 22 regional fetal medicine centres in England and Scotland, giving a response rate of 73% (16/22). Duplicate responses were obtained from two specialists in five centres. Where their answers differed, both specialists were required to come to a consensus on organisational and clinical questions. One respondent did not complete the question requesting a self‐assessment of confidence in managing pregnant patients with a history of FNAIT. Table 1 summarises the respondent demographics and experience with the management of FNAIT. Responses from five centres without regional fetal medicine centre designation were removed at the point of analysis.

**TABLE 1:** Demographic features of survey respondents.

Question (number of responses)	Response	*N* (%)
Physician speciality (21)	Haematology	10 (47.6)
	Obstetrician/fetal medicine	11 (52.4)
Who manages pregnant patients with a history of FNAIT in your hospital? (could select >1) (16)	Haematology	8 (50)
	Obstetrician/fetal medicine	14 (87.5)
	Neonatology/paediatrics	1 (6.3)
	No formal service	1 (6.3)
Number of new FNAIT cases at the site in the last 5 years (16)	0–3	5 (31.3)
	4–6	7 (43.8)
	7–10	1 (6.3)
	>10	3 (18.8)
Number of referrals for counselling for future pregnancies at the site in last 5 years (16)	0–3	6 (37.5)
	4–6	9 (56.3)
	7–10	0
	>10	1 (6.3)
Are all patients in the region referred to the regional fetal medicine centre? (16)	Yes	13 (81.3)
	No	3 (18.8)
Confidence managing pregnant patients with a history of FNAIT (20)	5 (highest confidence)	6 (30.0)
	4	13 (65.0)
	3	1 (5.0)
	2	0
	1 (lowest confidence)	0

Abbreviation: FNAIT, fetal and neonatal alloimmune thrombocytopenia.

The survey was answered by haematologists (10/21; 47.6%) and fetal medicine specialists (11/21; 52.4%) (Table 1). In most hospitals, fetal medicine specialists counselled women with previous FNAIT for future pregnancies (14/16; 87.5%) with haematology involved in half of the cases (8/16; 50%). In most instances, women with previous FNAIT were all referred to the regional fetal medicine centre (13/16; 81.3%). Ninety‐five per cent (19/20) of respondents rated their confidence managing FNAIT as high or very high (4/5 or 5/5).

The first four cases posed similar scenarios that differed only by antibody type and the presence (high risk) or absence (standard risk) of intracerebral bleeding in a neonate from a previous pregnancy. These are summarised in Table 2.

**TABLE 2:** Antenatal management of pregnant patients with a history of FNAIT.

Scenario		Scenario 1: Anti‐HPA‐1a standard risk	Scenario 2: Anti‐HPA‐1a high risk	Scenario 3: Anti‐HPA‐5b standard risk	Scenario 4: Anti‐HPA‐5b high risk
Medical management	No management required	0	0	2/16 (12.5%)	0
	IVIg weekly	14/16 (87.5%)	8/16 (50%)	13/16 (81.3%)	11/16 (68.8%)
	IVIg with steroids	2/16 (12.5%)	8/16 (50%)	1/16 (6.3%)	4/16 (25.0%)
	Unsure, would ask national specialist advice	0	0	0	1/16 (6.3%)
Starting dose of IVIg	0.5 g/kg/week	0	0	1/14 (7.1%)	0
	1 g/kg/week	16/16 (100%)	13/16 (81.3%)	13/14 (92.9%)	14/15 (93.3%)
	2 g/kg/week	0	3/16 (18.8%)	0	1/15 (6.7%)
	Other	0	0	0	0
Gestational age to start IVIg in weeks	12–16* ^,^ ^a^	4/16 (25.0%)	16/16 (100%)	1/14 (7.1%)	12/15 (80.0%)
	17–19	5/16 (31.3%)	0	3/14 (21.4%)	1/15 (6.7%)
	20–24 *, ^b^	7/16 (43.8%)	0	10/14 (71.4%)	3/15 (20.0%)
Consideration of FBS/IUT?	Yes	2/16 (12.5%)	3/16 (18.8%)	0	2/16 (12.5%)
	No	14/16 (87.5%)	13/16 (81.3%)	14/14 (100%)	14/16 (87.5%)
Consideration of escalated treatment?	Yes	2/16 (12.5%)	7/16 (43.8%)	2/16 (12.5%)	6/16 (37.5%)
	No	14/16 (87.5%)	9/16 (56.3%)	14/16 (87.5%)	10/16 (62.5%)
Mode of delivery	Delivery at 37 weeks: Vaginal or Caesarean section as per obstetric indications*	7/16 (43.8%)	6/16 (37.5%)	6/16 (37.5%)	4/16 (25.0%)
	Elective caesarean at 37 weeks*	8/16 (50%)	10/16 (62.5%)	9/16 (56.3%)	11/16 (68.8%)
	Induction of labour at 37 weeks*	0	0	0	0
	Elective caesarean at term	1/16 (6.3%)	0	1/16 (6.3%)	1/16 (6.3%)

Abbreviations: FBS, fetal blood sampling; FNAIT, fetal and neonatal alloimmune thrombocytopenia; IUT, intrauterine platelet transfusion; IVIg, intravenous immunoglobulin.

^a^ Recommendation for high‐risk pregnancies.

^b^ Recommendation for standard‐risk pregnancies.

* Points marked with an asterisk are strong recommendations in the international FNAIT consensus guidelines.

For standard‐risk pregnancies, most respondents agreed that patients with anti‐HPA1a [14/16 (87.5%)] or anti‐HPA5b antibodies [13/16 (81.3%)] should be treated with weekly IVIg without corticosteroids, and that escalation of the IVIg dose was unnecessary for either antibody type [14/16 (87.5%)]. There was complete consensus that the starting dose of IVIg should be 1 g/kg/week for patients with anti‐HPA1a antibodies [16/16 (100%)] and near‐complete agreement that the same dose should be used for anti‐HPA5b antibodies [13/14 (92.9%)]. The majority of respondents [10/14 (71.4%)] recommended initiating treatment for anti‐HPA5b–associated standard‐risk pregnancies between 20 and 24 weeks of gestation, while a smaller proportion [3/14 (21.4%)] advised starting between 17 and 19 weeks. There was low agreement (<50%) regarding the optimal gestational age to initiate IVIg for standard‐risk pregnancies with anti‐HPA1a antibodies, although all responses were prior to 24‐week gestation.

For high‐risk pregnancies, there was complete agreement that IVIg treatment should commence between 12 and 16 weeks of gestation for patients with anti‐HPA1a antibodies [16/16 (100%)] and substantial agreement for those with anti‐HPA5b antibodies [12/15 (80%)]. Half of respondents [8/16 (50%)] supported treatment with IVIg alone for anti‐HPA1a and 68.8% [11/16] for anti‐HPA5b antibodies, while a considerable proportion preferred a combination of IVIg and prednisolone for anti‐HPA1a [8/16 (50%)] and anti‐HPA5b [4/16 (25%)] antibodies. There was also moderate agreement that treatment escalation should not be considered in high‐risk pregnancies, with 56.3% [9/16] and 62.5% [10/16] recommending against escalation of treatment for anti‐HPA1a and anti‐HPA5b antibodies respectively. How treatment might be escalated was not defined in the survey.

Across all scenarios, fetal blood sampling and intrauterine platelet transfusion were generally considered unnecessary, with at least 80% of respondents recommending against their use. Most respondents advised delivery at 37‐week gestation for both standard‐ and high‐risk pregnancies associated with anti‐HPA1a and anti‐HPA5b [15/16 (93.7%) in both cases] antibodies, although there was variation in opinion as to whether delivery should follow obstetric indications or if a caesarean section should be mandated.

None of the respondents considered ‘no management’ to be appropriate for standard‐ or high‐risk pregnancies associated with anti‐HPA1a antibodies or for high‐risk pregnancies associated with anti‐HPA5b antibodies. Two respondents [2/16 (12.5%)] selected no management for a standard‐risk pregnancy with anti‐HPA5b antibodies.

For patients with anti‐HPA1a antibodies or those at risk of developing anti‐HPA1a antibodies but without a history of FNAIT, there was less consensus (Table 3). There was strong agreement (75%–100%) that partners of pregnant women who have a sister affected by FNAIT and are HPA1b1b should undergo HPA typing [16/16 (100%)], as should partners of women with an incidental finding of an anti‐HPA1a antibody [16/16 (100%)]. There was low concordance (less than 50%) for all other recommendations.

**TABLE 3:** Responses focusing on women at increased risk of FNAIT but without a personal history of FNAIT.

		Scenario 5: HPA1b1b pregnant woman with a sister affected by FNAIT. (*N* = 16), *N* %	Scenario 6: HPA1a antibodies found incidentally on platelet donation. (*N* = 16), *N* %
Management of pregnancy	Partner testing for HPA type only	6 (37.5%)	8 (50.0%)
	Partner testing for HPA type and increased ultrasound frequency	2 (12.5%)	4 (25.0%)
	Partner testing for HPA type and regular HPA1a antibody testing	1 (6.3%)	0
	Partner testing for HPA type and check human leukocyte antigen (HLA)‐DRB3*01:01 status	1 (6.3%)	1 (6.3%)
	Partner testing for HPA type and increased ultrasound frequency and regular HPA1a antibody testing	2 (12.5%)	0
	Partner testing for HPA type and check HLA‐DRB3*01:01 status and regular HPA1a antibody testing	1 (6.3%)	0
	Partner testing for HPA type and check HLA‐DRB3*01:01 status and regular HPA1a antibody testing and increased scan frequency	1 (6.3%)	0
	Partner testing for HPA type and empirical IVIG	2 (12.5%)	3 (18.8%)

Abbreviations: FNAIT, fetal and neonatal alloimmune thrombocytopenia; IVIg, intravenous immunoglobulin.

Responses related to treatment of a pregnant patient with a previous pregnancy consistent with FNAIT clinically, but with negative serological testing (including maternal–paternal crossmatch), was variable (Table 4). The majority agreed that no antenatal treatment was required [10/16 (62.5%)] with about 30% suggesting consulting with a national specialist [5/16 (31.3%)].

**TABLE 4:** Management of a patient with a clinical history consistent with FNAIT but with negative serological testing.

Scenario 7: Clinically consistent with FNAIT but negative serological testing (*N* = 16), *N* %		
Management of pregnancy	*No antenatal treatment required*	*10 (62.5%)* [4/10 (40%) recommended no antenatal treatment but more frequent antenatal ultrasound scans]
	Empirical treatment with IVIg	1 (6.3%)
	Unsure, would ask national specialist advice	5 (31.3%)

*Note*: Statements with 50%–74.9% concordance are marked in italics.

Abbreviation: FNAIT, fetal and neonatal alloimmune thrombocytopenia.

## DISCUSSION

This survey provides the first description of real‐world FNAIT practice in England and Scotland and highlights the extent of variation in practice. This survey shows consistency in several aspects of management and inconsistencies in others. Some consistency is expected and aligns with international guidelines and consensus, such as the use of IVIg to treat standard‐ and high‐risk pregnancies with anti‐HPA1a antibodies. In other areas, however, UK practice is consistent despite international variability; for instance across all scenarios, there was a strong preference for 1 g/kg/week of IVIg despite a range of doses being proposed in international guidelines. UK specialists also reported similar management approaches to pregnancies with anti‐HPA1a and anti‐HPA5b antibodies, which do not reflect the findings of the international Delphi consensus study. The survey also found areas of variability, most notably in how deliveries are managed and in scenarios involving pregnancies at risk but without a personal history of FNAIT. These inconsistencies reflect aspects of care where there is no international consensus and highlight underlying gaps in the evidence base.

For pregnancies with anti‐HPA1a antibodies, which account for most pregnancies at risk of FNAIT in the United Kingdom, reported management is largely consistent with international guidelines. This familiarity with guidance may also explain the high levels of confidence reported by specialists, 95% of whom rated their confidence as 4 or 5 out of 5. UK specialists indicated that they would treat both standard‐ and high‐risk pregnancies with anti‐HPA1a antibodies using antenatal IVIg. Among standard‐risk women, IVIg was initiated across a range of gestational ages but most commonly between 20 and 24 weeks, in line with guideline recommendations. For high‐risk pregnancies, respondents strongly favoured earlier initiation of IVIg, between 12 and 16 weeks of gestation. Although clinical evidence for this earlier timing is limited, it is biologically plausible and consistent with current international guidance. Fetal platelet HPA expression is thought to begin around 12‐week gestation,^19^ and an international cohort study reported that a significant proportion of ICHs occur in the second trimester, with 54% occurring before 28‐week gestation.^20^ Respondents were also consistent in not adding corticosteroids to IVIg for standard‐risk pregnancies, while their use in high‐risk pregnancies was variable, patterns that mirror findings from the Delphi consensus study.^18^

Some areas of UK practice were consistent despite the wide range of management approaches employed internationally. Our survey found strong consensus around an IVIg dose of 1 g/kg/week, whereas international guidelines propose multiple regimens (five for high‐risk pregnancies and three for standard‐risk pregnancies), and NHS England recommends two regimens in standard‐risk pregnancies, either 0.5 g/kg/week or 1 g/kg/week.^21^ Our findings indicate that the lower dose, 0.5 g/kg/week, is rarely used in England and Scotland. Despite the range of doses suggested in guidelines, this is perhaps not surprising given the limited evidence base. The only randomised trial directly comparing 0.5 g/kg/week to 1 g/kg/week in standard‐risk women included just 23 participants and was underpowered to demonstrate non‐inferiority of the lower dose. Nevertheless, there were no ICH events in either arm and no statistically significant differences in neonatal platelet count.^16^

Furthermore, consistent in the United Kingdom, despite international variability, is the finding that withholding antenatal IVIg in standard‐risk pregnancies is rarely practiced in England and Scotland. All specialists surveyed reported treating standard‐risk pregnancies with anti‐HPA1a antibodies using IVIg with or without steroids. While this approach aligns with practice in many countries, emerging evidence suggests that the benefit of IVIg may not be uniform and may depend on baseline risk. Recent data suggest that the risk of ICH in standard‐risk pregnancies is low, raising questions about the magnitude of benefit derived from routine use of IVIg in all standard‐risk pregnancies. Furthermore, the evidence base supporting IVIg is derived largely from observational studies and trials comparing IVIg‐based regimens rather than placebo‐controlled studies, and its efficacy in standard‐risk pregnancies remains uncertain. Notably, the ongoing FREESIA‐1 trial, evaluating the FcRn inhibitor nipocalimab versus placebo, represents the first placebo‐controlled randomised controlled trial in FNAIT and reflects a shift in opinion regarding the acceptability of placebo in standard‐risk pregnancies.^22^ These developments, alongside increasing support for risk‐stratified management approaches that reserve IVIg treatment for high‐risk pregnancies, highlight ongoing international debate regarding the routine use of IVIg in standard‐risk cases.^23^

Specialists in England and Scotland consistently reported similar management preferences for pregnancies with anti‐HPA1a and anti‐HPA5b antibodies. This is in line with current NHS England guidance^21^ but contrasts with the Delphi consensus, in which there was no agreement on the management of subsequent pregnancies after FNAIT with anti‐HPA5b antibodies; most respondents opted against IVIg unless there was a history of fetal or neonatal ICH.^18^ This lack of agreement likely reflects ongoing uncertainty regarding the clinical significance of anti‐HPA5b antibodies.

Anti‐HPA5b antibodies are present in approximately 2% of healthy pregnant women and occur at a similar frequency in pregnancies investigated for suspected FNAIT (1.96% vs. 3.4%), suggesting that their detection in such cases may be coincidental rather than causative.^3^ Furthermore, a Swedish registry study of neonates born with ICH identified anti‐HPA5b antibodies in only 1.9% of cases, consistent with the expected background prevalence and supporting a coincidental association.^24^

Interpretation of retrospective studies in suspected FNAIT cohorts is limited by selection and indication bias, as cases are typically identified following adverse outcomes such as ICH. In the retrospective series by de Vos et al., severe bleeding was reported in 4/40 (10%) of anti‐HPA5b‐positive pregnancies with an incompatible fetus; however, ICH was also observed in 1/7 (14%) anti‐HPA5b‐positive pregnancies with a compatible fetus, indicating that the presence of these antibodies does not necessarily imply causation.^1^ Taken together, these findings highlight the limitations of using antibody detection alone to infer causality and suggest that there is currently insufficient evidence to support a causal role for anti‐HPA5b antibodies in fetal ICH.

Our study has several limitations. Although it used a multidisciplinary approach, incorporating expert input from fetal medicine specialists and haematologists experienced in FNAIT management, the sample size was relatively small. In five centres where more than one clinician responded, respondents were asked to agree a single consensus answer. This approach may have introduced bias, potentially bringing reported practice more closely in line with guideline recommendations than in centres with only a single respondent. Furthermore, the use of clinical scenario questions cannot fully capture the complexity and nuance of real‐world clinical decision‐making. As a result, the answers given may not always mirror actual practice, particularly in challenging or atypical cases.

Despite these limitations, the high response rate to the survey means that participating centres represent 73% of regional fetal medicine centres in the United Kingdom, supporting the generalisability of the findings. In addition, the use of structured clinical scenarios allowed us to explore management in areas where the evidence base is weak and formal guidance is limited, particularly in relation to incidental HPA antibody findings and the management of sisters of patients with FNAIT. These scenarios helped to identify specific domains in which clinicians lack clear evidence‐based direction and may therefore benefit most from future research and guideline development.

Overall, this survey suggests that UK practice sits somewhere in the middle of the multiple acceptable treatment strategies described in international guidelines. Survey respondents tended to treat, rather than not treat, in scenarios where international guidance and consensus are less prescriptive (notably pregnancies with anti‐HPA5b antibodies), and used IVIg at an intermediate dose rather than lower (0.5 mg/kg/week) or higher (2 g/kg/week) regimens. Corticosteroid use was variable in high‐risk pregnancies and consistently not used in standard‐risk pregnancies. These findings underline the need for robust comparative data to inform optimal dosing, timing and patient selection and provide a foundation for future efforts to standardise and optimise FNAIT care in England and Scotland.

## AUTHOR CONTRIBUTIONS

AA, WT, EV and MJRD designed the study. BH built the survey on the survey platform. AA and MJRD analysed the data. AA, CP and MJRD drafted the paper. MM, LL, JBB and MJRD critically revised the paper.

## FUNDING INFORMATION

The study was supported by NHS Blood and Transplant, United Kingdom.

## CONFLICT OF INTEREST STATEMENT

JBB consultants for Johnson and Johnson, Argenx, UCB and RallyBio.

## Supporting information


Appendix 1.

Appendix 2.

## Data Availability

The data that support the findings of this study are available from the corresponding author upon reasonable request.
