# Supplementary material for: Fetal and neonatal alloimmune thrombocytopenia (FNAIT): Survey of UK fetal medicine centres on antenatal management of subsequent affected pregnancies
Source: Br J Haematol. 2026 May 19;209(1):240–7. doi: 10.1111/bjh.70564 (PMC13340498; doi:10.1111/bjh.70564)
Supplement: Supplementary file 1 — Appendix 1. [file BJH-209-240-s002.docx]

**Appendix 1: Survey questions**

**Scenario 1. Anti- HPA1a, Standard risk:** A patient carries a fetus who is born without complications at term. Spontaneous bruising is noted, and a full blood count shows a platelet count of 35 x 10^9^/L. The cranial ultrasound is normal. The mother has anti-HPA1a antibodies, and the father is homozygous for HPA1a. She presents at 11 weeks gestation two years later, pregnant from the same partner.

**Scenario 2. Anti- HPA1a, High risk:** A patient reports loss of fetal movements at 32 weeks gestation. The fetus is found to have intracerebral bleeding on imaging, leading to an intrauterine death. The mother has anti-HPA1a antibodies, and the father is homozygous for HPA1a. Cordocentesis before fetal death shows the fetus had a platelet count of 7 x 10^9^/L, and was heterozygous for HPA-1a.

**Scenario 3. Anti- HPA5b, Standard risk.** A patient carries a fetus who is born without complications at term. Spontaneous bruising is noted, and a full blood count shows a platelet count of 35 x 10^9^/L. The cranial ultrasound is normal. The mother has anti-HPA5b antibodies, and the father is homozygous for HPA5b. She presents at 11 weeks gestation, two years later, pregnant from the same partner.

**Scenario 4.** **Anti-** **HPA5b, High risk.** A patient carries a fetus who is born at term. Spontaneous bruising is noted, a full blood count reveals a platelet count of 15 x 10^9^/L. The cranial ultrasound shows evidence of intracerebral haemorrhage. The mother has anti-HPA5b antibodies, and the father is homozygous for HPA5b.

**Scenario 5:** A patient is diagnosed with FNAIT with anti-HPA1a antibodies. Her neonate had an intracerebral haemorrhage but has made a good recovery. Her sister was tested, and is found to be homozygous for HPA1b1b. The previously unaffected sister is referred to you at 8 weeks gestation into her first pregnancy.

**Scenario 6:** A woman is informed by the blood service when she tries to donate platelets that she has anti-HPA1a antibodies. She is now 10 weeks of gestation into her first pregnancy.

**Scenario 7**: A patient reports loss of fetal movements at 32 weeks of gestation. The fetus is found to have intracerebral bleeding on imaging, leading to intrauterine death. Cordocentesis before fetal death shows the fetus had a platelet count of 7 x 10^9^/L. The mother, father and fetus all have an HPA 1a1b 5a5b genotype. Direct platelet crossmatch and testing for anti-HPA antibodies, including Luminex bead testing, are negative. She presents in her subsequent pregnancy.
