# Supplementary material for: Fetal and neonatal alloimmune thrombocytopenia (FNAIT): Survey of UK fetal medicine centres on antenatal management of subsequent affected pregnancies
Source: Br J Haematol. 2026 May 19;209(1):240–7. doi: 10.1111/bjh.70564 (PMC13340498; doi:10.1111/bjh.70564)
Supplement: Supplementary file 2 — Appendix 2. [file BJH-209-240-s001.docx]

**Appendix 2: Acknowledgements**

Will Thomas – Department of Haematology, Cambridge University Hospitals NHS Foundation Trust, Cambridge, United Kingdom

Roobin Jokhi – Fetal Medicine Unit, Sheffield Teaching Hospitals NHS Foundation Trust, Sheffield, United Kingdom

Gill Swallow – Department of Haematology, Nottingham University Hospitals NHS Trust, Nottingham, United Kingdom

Rajeswari Parasuraman – Wessex Fetal Medicine Unit, University Hospitals Southampton NHS Trust, Southampton, United Kingdom

Umber Agarwal – Fetal Medicine Unit, Liverpool Women’s NHS Foundation Trust, Liverpool, United Kingdom

Suzanne Docherty – Department of Haematology, Norfolk and Norwich University Hospitals NHS Foundation Trust, Norwich, United Kingdom

Victoria Bills – Fetal Medicine Unit, University Hospitals Bristol NHS Foundation Trust, Bristol, United Kingdom

Shona Cowan - Fetal Medicine Unit, NHS Lothian, Edinburgh, United Kingdom

Amy Webster – Department of Haematology, University Hospitals of Leicester NHS Trust, Leicester, United Kingdom

Surabhi Nanda – Fetal Medicine Unit, Guys and St Thomas’ NHS Foundation Trust, London, United Kingdom

Evangelina Vlachodimitropoulou – Department of Obstetrics and Gynaecology, King’s College London, London, United Kingdom

Michael Desborough – Department of Clinical Haematology, Oxford University Hospitals NHS Foundation Trust, United Kingdom

Mike Murphy - Department of Clinical Haematology, Oxford University Hospitals NHS Foundation Trust, United Kingdom

Gill Lowe – Department of Haematology, University Hospitals Birmingham NHS Foundation Trust, Birmingham, United Kingdom

Leo Gurney – Fetal Medicine Unit, Birmingham Women’s and Children’s NHS Foundation Trust, Birmingham, United Kingdom

Tracy Hui – Haematology Department, Imperial College Healthcare NHS Trust, London, United Kingdom

Christoph Lees – Centre for Fetal Care, Queen Charlotte’s and Chelsea Hospital, Imperial College Healthcare NHS Trust, London, United Kingdom

Jennifer Laird – Department of Transfusion Medicine, Patient Services, Scottish National Blood Transfusion Service, Glasgow, United Kingdom

Catherine Bagot - Department of Haematology, NHS Greater Glasgow and Clyde, Glasgow, United Kingdom

Andrew Breeze – Fetal Medicine Unit, Leeds Teaching Hospitals NHS Trust, Leeds, United Kingdom

Thomas Everett - Fetal Medicine Unit, Leeds Teaching Hospitals NHS Trust, Leeds, United Kingdom
